# Why do people donate to conservation? Insights from a ‘real world’ campaign

**DOI:** 10.1371/journal.pone.0191888

**Published:** 2018-01-25

**Authors:** Diogo Veríssimo, Hamish A. Campbell, Simon Tollington, Douglas C. MacMillan, Robert J. Smith

**Affiliations:** 1 Durrell Institute of Conservation and Ecology, School of Anthropology and Conservation, University of Kent, Canterbury, United Kingdom; 2 Research Institute for the Environment and Livelihoods, Charles Darwin University, Darwin, NT, Australia; 3 North of England Zoological Society, Chester Zoo, Chester, United Kingdom; Charles University, CZECH REPUBLIC

## Abstract

Non-governmental organisations (NGOs) play a key role in biodiversity conservation. The majority of these organisations rely on public donations to fund their activities, and therefore fundraising success is a determinant of conservation outcomes. In spite of this integral relationship, the key principals for fundraising success in conservation are still guided by expert opinion and anecdotal evidence, with very few quantitative studies in the literature. Here we assessed the behaviour of monetary donors across twenty-five different species-focused conservation campaigns organised by an NGO conservation and environmental society. The Australian Geographic Society (AGS) carried out fundraising campaigns over a five and half year period using an identical methodology in thirty-four of its country-wide network of outlet shops. AGS owns and operates these shops that sell toys and games related to science and nature. We tested how the following factors influenced monetary donations from members of the public:1) campaign duration, 2) appeal and familiarity of species, 3) species geographic distribution relative to the fundraising location, 4) level of income and education of potential donors, 5) age and gender profile of potential donors. Contrary to past research, we found most of these factors did not significantly influence the amount of donations made to each campaign by members of the public. Larger animals did elicit a significantly higher amount donated per transaction than smaller animals, as did shops located in poorer neighbourhoods. Our study findings contrast with past research that has focused largely on hypothetical donations data collected via surveys, and demonstrates the complexity and case-specific nature of relationships between donor characteristics and spending patterns. The study highlights the value of assessing real-world fundraising campaigns, and illustrates how collaboration between academia and NGOs could be used to better tailor fundraising campaigns to maximise donations from individual citizens.

## Introduction

Non-governmental organisations (NGOs) have been at the forefront of the conservation movement since the early 1900s [1]. These organisations generally rely on donations from the public to fund conservation programs [2]. For example, the World Wide Fund For Nature (WWF) and The Nature Conservancy, the world’s largest conservation NGOs, reported in 2014 that their largest single revenue source were donations from individual citizens, accounting for 55% and 37% of their respective income [3, 4]. Thus, the success of NGO fundraising efforts significantly impact conservation outcomes.

Fundraising has traditionally been considered an art rather than a science within the NGO sector. Few studies have been undertaken upon donor behaviour [5], and a survey of 136 health, arts and development NGOs across the UK, USA and Australia revealed that fewer than one third engaged in any marketing research, and only one percent considered it an important area of marketing [6]. This is a missed opportunity because most NGOs collect a wealth of relevant data through their accounting and donation systems, and NGOs do have greater fundraising success when they have undertaken market research [7, 8]. In the environmental sector, there are very few published accounts of market research [8], and therefore these NGOs rely mostly on anecdotal evidence to guide their fundraising strategies.

An example of this research gap is the selection and use of conservation flagships, which are species, biological groups, ecosystem or other entities used for conservation marketing activities [9] such as fundraising. Over the last decade there has been a shift in how conservation flagships are selected, with a stronger emphasis on understanding how different traits impact the attitudes of the target audiences [10–13]. The majority of studies have, however, focused on the role of flagships in the context of community-based conservation, where the objective is to influence the behaviour of a group of people towards more environmentally friendly alternatives. There have been a few studies investigating the impact of conservation flagships on fundraising [e.g. 14, 15] but they have largely focused on one or a small group of species and used metrics based on hypothetical donations [16]. The focus on single or small groups of species has made it difficult to understand which flagship traits matter to prospective donors. In addition, the focus on hypothetical donations (“stated preferences”), rather than actual donor behaviour (“revealed preferences”) [17] has made the data more vulnerable to biases. This is because most studies comparing hypothetical and actual donations find that hypothetical donations commonly over-estimate the amount the target audience is willing to donate [18, but see also 19].

In this study, we sought to address this fundraising and conservation knowledge gap by focusing on the drivers of donor behaviour for an environmental NGO. We used actual donation data gathered by the Australian Geographic Society (AGS), an NGO dedicated to supporting scientific research and protecting environmental heritage at the national level. The AGS runs standardised fundraising flagship campaigns through its chain of shops spread across Australia, which sell toys and games related to science and nature. These are passive fundraisers based on shop customers leaving donations at the till, a type of campaign used mostly by small and medium sized NGOs because they often have no dedicated fundraising staff [20].

This context provides the opportunity to investigate the influence of: seasonal fluctuations in spending patterns; the traits of the conservation flagship used, and; the demographic and socio-economic characteristics of the area where the fundraising campaigns took place. In line with this, we tested the following five hypotheses. The first is that campaign duration is positively associated with revenue. Campaign duration is intuitively seen as a key aspect of a marketing strategy, but no research exists in the environmental non-profit sector to understand its true importance [21, 22]. The second is that AGS customers prefer to donate to appealing large-bodied mammals over other species. While there is an extensive body of conservation literature suggesting that public attitudes are most positive towards mammalian megafauna [14, 23, 24], this claim has yet to be tested in the context of an actual conservation fundraising campaign. The third is that AGS customers prefer species found locally over those distributed further away. Familiarity is assumed to be an important factor in flagship success, given its importance in commercial brand marketing; this is mostly supported in the conservation context by attitudinal and stated preference data [25, 26] and has not been tested in a fundraising context. The fourth is that shops located in areas with higher levels of income and formal education receive more donations, and the fifth is that donation levels depend on the age and gender profile of the people living in the areas in which the shops are located. These latter two hypotheses have their genesis in the long running debate on the impact of donor characteristics, such as income, education, gender and age, in determining fundraising appeal effectiveness [27–30]. While much has been reported about the importance of these factors, there is little research existing that describes actual fundraising campaigns for environmental conservation.

## Methods

**Fig 1:**
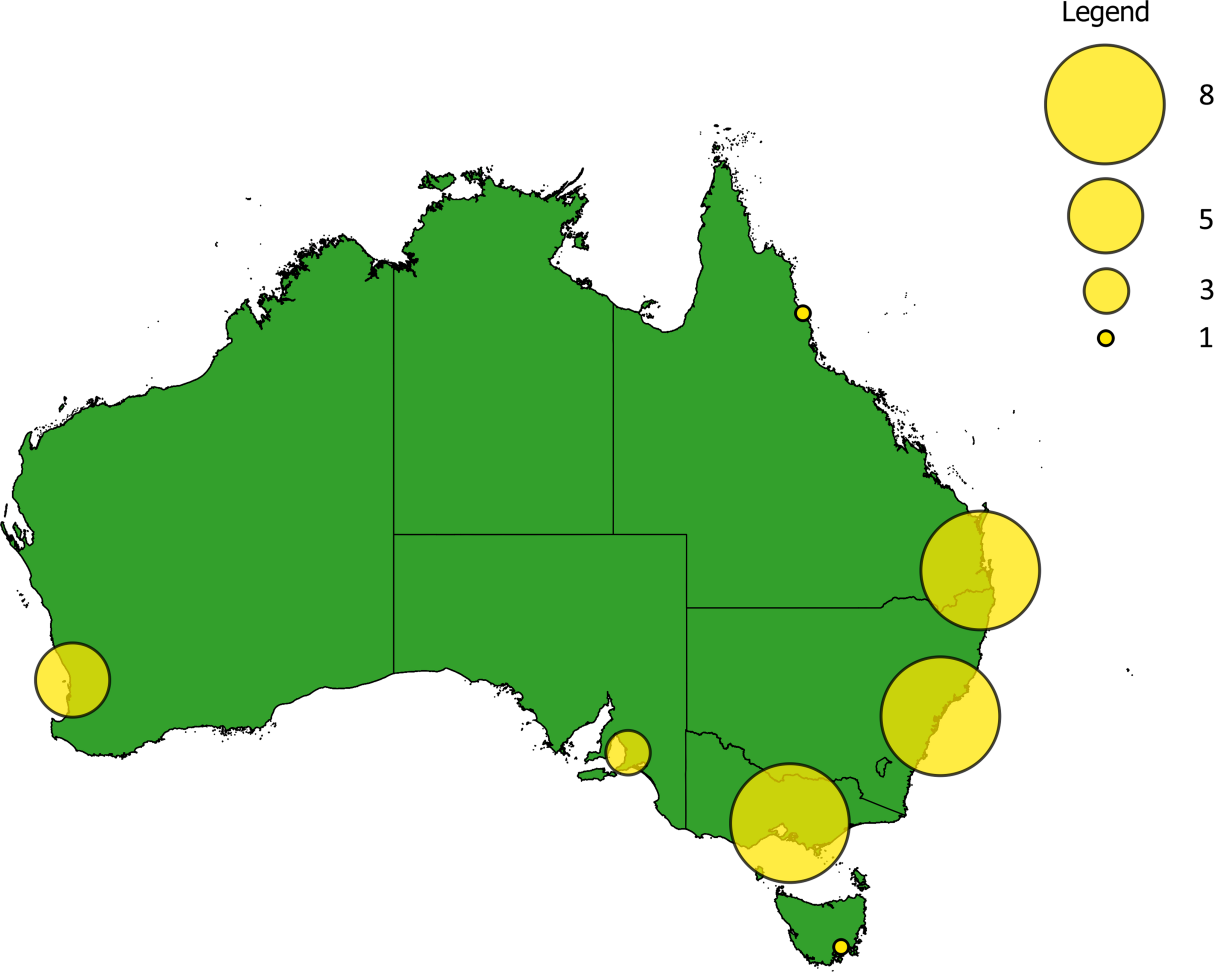
Location of the 34 shops of the Australian Geographic Society where flagship campaigns were run between January 2008 and June 2013. Larger circles indicate higher number of shops. Generated using QGIS 2.14.3 and open access data from http://biogeo.ucdavis.edu.

Between January 2008 and June 2013 the AGS ran 25 different fundraising campaigns in its shops (S1 Fig). Campaigns lasted a median of 2.5 months, with a minimum of one and a maximum of six months. For our analyses we only used data from shops that carried out all of these campaigns, excluding those that opened or closed for business during the 66-month period. This resulted in a sample size of 34 shops located across Australia, concentrated around major urban areas, and a total sample size of 850 campaigns (Fig 1). Each campaign focused on a specific topic concerning either a single species, habitat or conservation issue and was run simultaneously across all shops, with the same marketing materials, start and end date (S1 Fig). This involved placing an A3 poster describing the campaign next to a donation box of approximately 40 x 20 x 20 cm at the cash register counter in each AGS shop, thus relying on customers to read and interpret the information provided before deciding whether to donate and how much. Flyers with information detailing each campaign were placed next to the donation box, and the same imagery and information was placed in an advert in the Australian Geographic magazine.

The campaign topics were selected according to the strategic priorities of the AGS, often reflecting specific projects in need of funding at the time. The AGS shops sell toys and games related to science and nature, with consumer research showing their typical customer is 30 to 50 years old, middle class, and in the shop to buy gifts (AGS Marketing Department, personal communication).

### Donation data

We obtained total campaign revenue by summing the donations received in each shop during each campaign and converting this amount to 2013 Australian Dollars to account for inflation. Given that campaigns on different topics were run for different periods of time, and that footfall is unlikely to be consistent throughout the year, we incorporated shop-level data on campaign duration and number of customer transactions during each fundraising campaign. We also included a variable to account for the order in which the campaigns were conducted, given that the proportion of Australians donating to charity and the average donation have steadily increased in the last two decades [31].

### Campaign flagship trait data

For the conservation flagship trait data we focused on flagship type, body mass, appeal and familiarity, because these traits are known to influence the preferences of the public and the selection of conservation flagship by conservation NGOs [23, 32–34]. We obtained the data on species body mass from the PanTHERIA and Animal Diversity Web databases [35, 36]. We obtained data on species appeal and familiarity through an online survey, using the same photos that the AGS used in their marketing materials (magazine advert, posters and flyers). The survey was disseminated through the AGS Facebook and Twitter accounts (n = 122, only respondents resident in Australia were considered). Each respondent was presented with photos of ten of the species displayed in a random order and asked to “drag and drop” the photos to rank them from most to least appealing. To measure familiarity, respondents were asked if they had ever seen (in the wild, in captivity, in a book, TV, etc.) any of those species. We then calculated the appeal score as the average rank for each species, and familiarity as the percentage of respondents familiar with each species. Detailed survey results are presented in S1 and S2 Tables. This research was approved by the Ethics Committee of the School of Anthropology and Conservation of the University of Kent, UK. Informed consent was obtained from respondents.

We also considered the proximity of each species’ range to the location of each shop, to assess if customers were more willing to donate to species found locally [37]. The distribution maps provided by the IUCN Red List [38] were used to classify a species as “local” if its distribution included the Australian state where a specific shop was located.

### Demographic and socio-economic data

We obtained data from the 2011 Australian census on the gender, age, economic status, and educational profile of people living within the area surrounding each shop [39]. This was done at the level of Statistical Area 2 of the Australian Statistical Geography Standard, areas comprising 3,000 to 25,000 people, by using Google Earth to overlay the spatial census data with the locations of the AGS shops.

### Data analysis

We performed extensive exploratory analyses on our dataset by checking all the variables for heterogeneity of variance, residual normality and by visually inspecting them to identify potentially influential data points. We also investigated the explanatory variables for multicollinearity using variance inflation factors [40] and examined the relationships between them graphically using pair plots. Initial investigation involved characterising the monthly variation in customer number, to detect any anomalies such as the Christmas period, and to identify any obvious effect of campaign duration on the total funds raised.

We used generalised linear mixed-effect models (GLMMs) to describe the predictors of revenue and an information theoretic approach to assess model suitability based on the application of Akaike’s information criteria [41]. To account for any statistical non-independence of sampling the same shops and campaign topics, these were maintained as random effects in the models. The continuous predictor variables were centralised by standardising to a mean of zero and a standard deviation of 0.5 [42]. Model fit and parameter importance was assessed using the ‘dredge’ function of the multi-model inference package MuMIn for R [43], and a threshold of ΔAICc < 4 was used to select models for averaging [40]. We also assessed model fit by computing the estimated marginal R-squared values for each candidate model by using the appropriate function in the MuMIn package [42]. Furthermore, we calculated the weighted, absolute t-statistics across each of our candidate model sets in order to characterise relative variable importance [44, 45].

To test our first hypothesis, that the duration of campaigns positively predicted total revenue, we used the total funds raised per campaign or ‘revenue’ as the response variable and log-transformed it to improve residual normality. Two explanatory variables of campaign ‘duration’ and ‘time’ (reflecting the chronological order of campaigns) were included as fixed effects.

Revenue per customer transaction was used as the response variable to investigate our remaining hypotheses associated with the characteristics of individual-based donation behaviour. To investigate the effects of flagship traits on campaign revenue (hypotheses two and three), the model included the following fixed covariates: average species body mass, familiarity, appeal, location, time and flagship type. A full, descriptive list of explanatory variables can be found in Table 1. For the campaign that focussed on coral reefs, we used data for the species used in the campaigns promotional material, the clown fish (*Amphiprion ocellaris*). Lastly, we examined our fourth and fifth hypotheses on the effects of socio-economic characteristics using the explanatory variables for education and income and the demographic traits age and gender (Table 1). Campaigns with flagships that were not focused on the conservation of a biological unit (under the category “projects” in Table 1) were included in the analysis to test the first hypothesis but excluded from the analyses that followed. We carried out all our analyses using the R programming language [46] and constructed the mixed models using the ‘lme4’ package [47].

**Table 1:** Variables used in analysis to understand drivers of donations to fundraising campaigns run by the Australian Geographic Society.

Name	Definition
Revenue	Total revenue of a campaign, corrected for inflation using the inflation calculator of the Australian Bureau of Statistics. Continuous variable
Customers	Total number of transactions in a shop during a fundraising campaign. Continuous variable
Shop	Unique identifier for each shop. Categorical variable
Campaign	Unique identifier for each conservation campaign. Categorical variable
Time	Order in which the fundraising campaigns took place. Continuous variable
Duration	Number of months a fundraising campaign lasted. Continuous variable
Income	Index of Economic Resources score of residents in the area where a shop was located (Statistical Area 2 level of the Australian Statistical Geography Standard). This is a measure of access to economic resources. Continuous variable
Education	Index of Education and Occupation score of residents in the area where a shop was located (Statistical Area 2 level of the Australian Statistical Geography Standard). This is a measure of the education and occupation status of the residents. Continuous variable
Age	Median age of residents living in the area where the shop was located (at the Statistical Area 2 level of the Australian Statistical Geography Standard). Continuous variable
Gender	Proportion of female residents in the area where the shop was located (at the Statistical Area 2 level of the Australian Statistical Geography Standard). Continuous variable
Flagship type	Topic of the fundraising campaign, divided into the following categories: mammal, bird, amphibian, reptile, fish, biological group, ecosystem or a project flagship (e.g. seed bank). Categorical variable
Body mass	Average individual mass of the species targeted by fundraising campaigns [35, 36]. Continuous, log-transformed variable [23]
Familiar	If a given fundraising campaigns targeted a familiar species (as defined by more than 50% of respondents recognizing the species). Binary variable
Appeal	If a campaign focused on an appealing species (as defined as an average ranking of 5 or less, out of 10). Binary variable as not all campaigns targeted species
Location	If the flagship type of a fundraising campaign existed in the Australian state where a shop was located. Binary variable

## Results

Of the 25 campaigns, 20 focused on a single species, half of which were mammals, with the remaining including birds, reptiles, amphibians and fish. The other five campaigns focused on invertebrates, coral reefs and on non-biodiversity topics, such as an effort to rescue animals from a flood or the construction of a historical building replica.

Likelihood-ratio chi-squared tests revealed that both duration (β = 0.40, SE = 0.19, χ^2^ = 4.45, p = 0.03) and time (β = 0.08, SE = 0.02, χ^2^ = 9.62, p<0.01) were important determinants of total amount fundraised by a given campaign. However, model checking revealed the existence of a potentially outlying data point. A single campaign lasted for six months whilst all others were run for between one and three months and this single data point proved influential. Omitting these data from the analysis revealed that duration was not a significant predictor of total revenue (β = 0.47, SE = 0.40, χ^2^ = 1.52, p = 0.22). Time, or the chronological order of campaigns, remained an important positive predictor of total amount fundraised by a given campaign in this re-worked analysis (β = 0.09, SE = 0.03, χ^2^ = 6.71, p<0.01). Time accounted for approximately 9% of the total variance in log revenue according to marginal R^2^ values. Given that time appeared to be an important predictor in total revenue raised it was included as a fixed covariate in our subsequent analyses.

**Fig 2:**
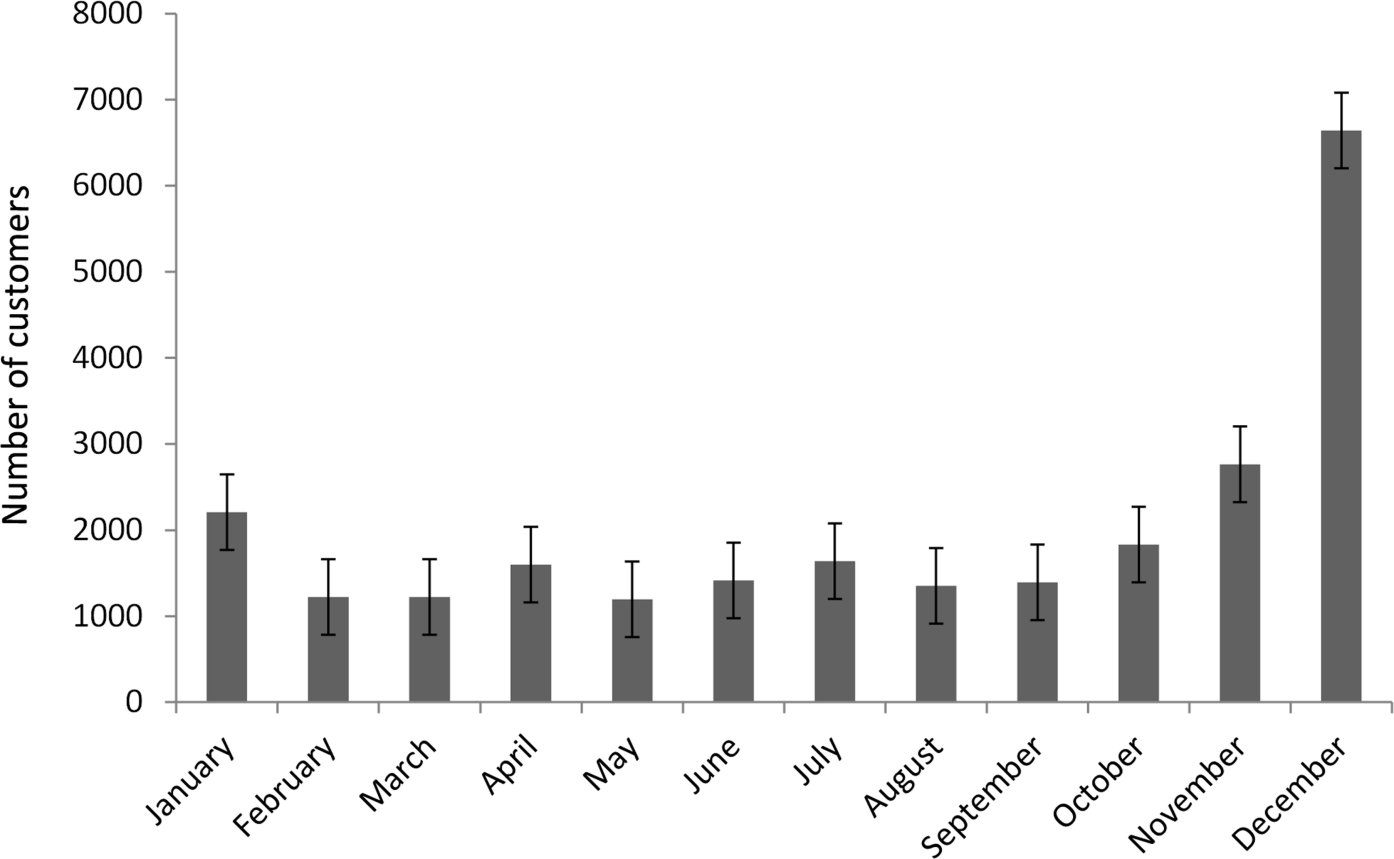
Mean number of customers (± SE) per shop by month.

**Fig 3:**
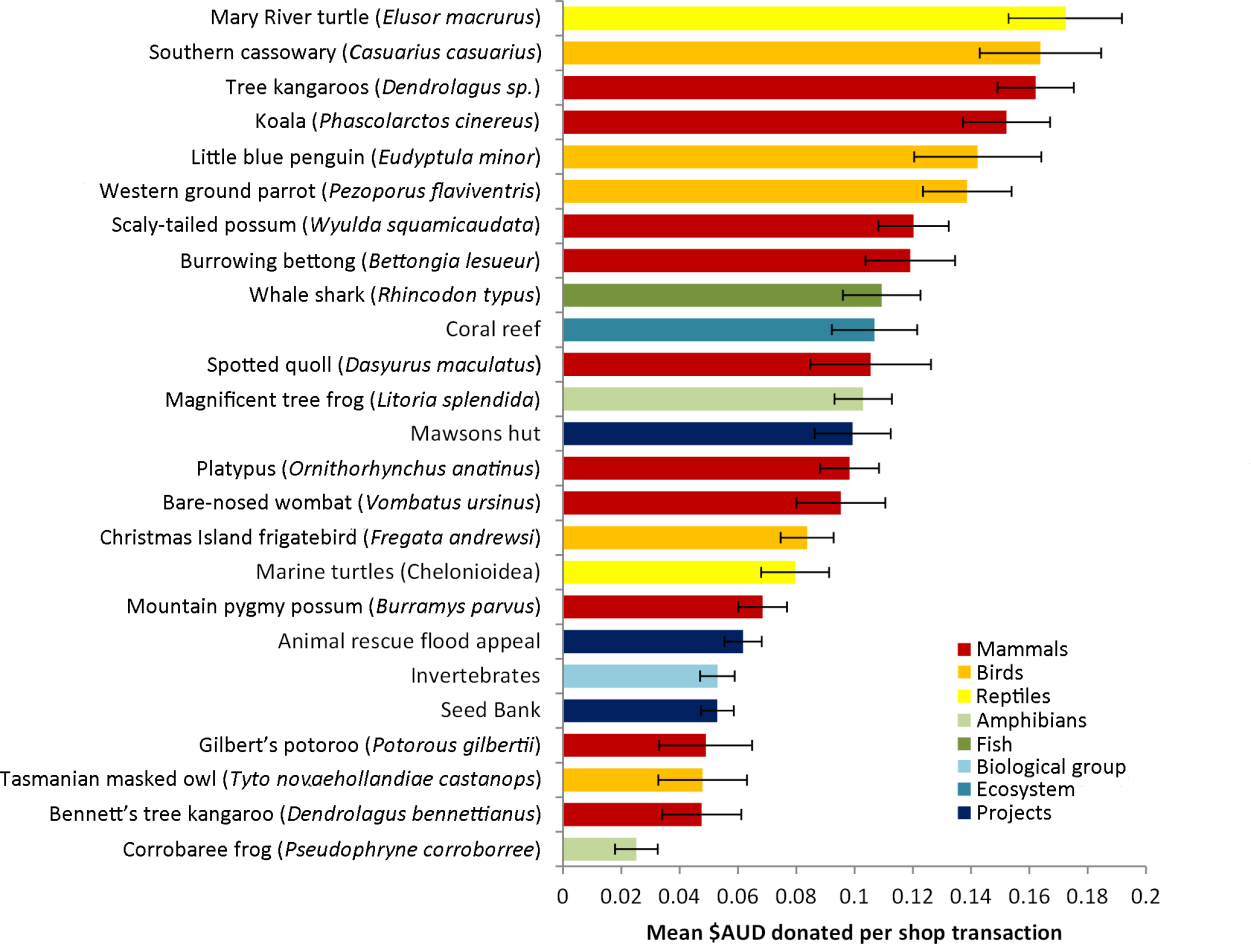
Mean revenue (± SE) raised per customer for the different conservation fundraising campaigns.

The number of customers per shop was highly seasonal, with the Christmas period being the busiest (Fig 2). To account for this variation and to investigate individual donation behaviour, the (logged) mean amount donated per customer transaction was used as the response variable in all our further analysis. This number was obtained by dividing the total amount donated to a given campaign in a given shop by the number of transactions, including those transactions that did not include a donation. The revenue per transaction raised by each fundraising campaign differed markedly, with the campaign for the Mary River turtle (AUS$ 0.17) raising nearly six times more per customer transaction than that of the Corrobaree frog (AUS$ 0.03; Fig 3).

Model selection to investigate the importance of flagship characteristics on individual donor behaviour (hypotheses two and three) revealed only two models at ΔAICc < 4 (S3 Table). Only two of our variables of importance, body mass and time were retained, both positively predicting revenue per transaction. The top model featured only time as an important predictor with a marginal R^2^ value of 0.15, while the second, with a marginal R^2^ value of 0.19, featured variables of time and body mass (S3 Table).

Model averaging revealed that time was the strongest predictor of revenue per transaction (time t-statistic = 4.45, body mass t-statistic = 0.57) suggesting that although there was a tendency for people to donate more money to larger bodied species (partially supporting hypothesis two), donations per transaction significantly increased over time regardless of the campaign topic (Table 2).

**Table 2:** Model-averaged estimates for generalised linear mixed models coefficients (β), standard errors (SE) and confidence intervals for conservation flagship related drivers of donations to the AGS fundraising campaigns. Only candidate models at ΔAIC < 4 candidate set were considered. Model averaged coefficients are ranked for relative importance using weighted absolute t statistics.

Variable	β	SE	Lower 95% CI	Upper 95% CI	Absolute t-values
Intercept	0.29	0.02	0.26	0.32	
Time	0.11	0.02	0.06	0.16	4.45
Body mass	0.05	0.02	0.01	0.10	0.57

Model selection to investigate the socio-economic and demographic characteristics of customers in the area surrounding a shop (hypotheses four and five), revealed only two models where ΔAICc < 4 (S4 Table). The top model containing both time and income, revealed a marginal R^2^ value of 0.19. The second contained only time and had a marginal R^2^ value of 0.15.

Model averaging identified time as the most important predicting variable (time t-statistic 4.34, income t-statistic 1.59) reflecting a similar pattern to our previous model (Table 3). Income was negatively associated with revenue per transaction indicating that customers in less wealthy areas donated more money per transaction.

**Table 3:** Model-averaged estimates for generalised linear mixed models coefficients (β), standard errors (SE) and confidence intervals for socio-economic and demographic drivers of donations to the AGS fundraising campaigns. Only candidate models in the ΔAIC < 4 candidate set were considered. Model averaged coefficients are ranked for relative importance using weighted absolute t statistics.

Variable	β	SE	Lower 95% CI	Upper 95% CI	Absolute t-values
Intercept	0.29	0.02	0.26	0.32	
Time	0.11	0.02	0.06	0.16	4.34
Income	-0.05	0.02	-0.09	-0.02	1.59

## Discussion

NGOs are a vital component of the conservation movement [48] but their continued survival and resultant impact depends on their ability to raise money from the public. In this study, we used monetary donations collected by a conservation-focused NGO to examine the drivers of donations for a fundraising campaign. Here, we discuss what variables influenced donor spending, and found that in contrast to stated preference survey data, only species body weight and donor income were linked to mean amount donated per transaction, with larger species and poorer areas being linked to higher donations. We explain these differences in terms of the characteristics of the AGS campaign, and argue that our study, based on actual donation data collected in a real-world setting, might be more relevant than previous research based on hypothetical survey data. We also highlight the insights that our research can give to similar fundraising campaigns (i.e., those focused on generating small donations from a large number of private individuals) and finish by advocating for more extensive collaboration between academia and NGOs, by arguing that this is the best way to encourage more marketing research in the conservation NGO sector.

### Flagship impact

We tested whether conservation flagship traits influenced donations and were surprised to find that in the context of the species traits considered in this study, only body mass had an influence, albeit small, on the amount donated per customer, as documented in several prior studies [23, 49]. This result is surprising as the variables used in this research had previously been found to be important in a variety of past studies [24, 50–53]. One reason for this unexpected difference could have been the characteristics of the AGS fundraising campaign, as this relied on shoppers reading and interpreting the information provided and then putting money in a donation box by the cash register. Such passive fundraising schemes are characterised by low donor involvement, which means people’s behaviour is more influenced by the shopping experience than by the information provided [54]. In fact, evidence from previous studies [54] suggests some donors completely ignore the information provided and donate money irrespective of the specific cause. This echoes findings from the commercial sector, where many purchases occur without obvious evidence that any information processing has taken place [55]. This scenario is also supported by the small amounts donated per customer, as the highest campaign mean value was around AUS$0.2. In such circumstances, the decision to donate could be determined chiefly by the donors’ attitude towards the fundraising organization [56, 57]. Under this model, the perception of the AGS target audience might have trumped any impact on the variation in conservation flagships. This could be particularly important in this study, as AGS customers are likely to have a positive image of AGS as an institution, making the campaign topic less influential.

Alternatively, the limited influence of the flagships’ traits may be because the specific target audience genuinely has no strong preference or because the species traits so far highlighted in the conservation flagship literature, on which our model is based, failed to explain the preferences of the AGS. If the former is true, it could have important implications. For example, this apparent indifference of AGS customers as to whether the flagship is familiar suggests that even species unknown to the target audience could be used as flagships, and that raising the profile of a flagship before the campaign would not increase fundraising revenue. Similarly, the lack of interest in whether the flagship was local (i.e. lives in the same state), suggests there would be little benefit in tailoring the fundraising campaigns to have more regionally-specific flagships. Thus, further research is needed to investigate the potential role of flagships in this context, perhaps by looking not only at a wider range of flagship traits, and other target audience groups but also whether results would be improved by using flagship fleets, representing the flagship in a more anthropomorphised way [12, 58], or using ecosystems or protected areas as flagships [9, 59].

Future research should also be designed to overcome the limitations of our study that resulted from using data from real-world campaigns, which were not primarily intended for analysis. This future research should use a range of different campaigns for each ecological group (our analysis only had one campaign based on a fish or ecosystem). This could be achieved by using more than one image type per campaign to ensure that the photo used did not have an impact, and randomised when each campaign was run in each shop to better tease out the effect of seasonality and flagship type. It is also worth noting that given the retail context of our study, there is potential for the range of products available in-store to generate priming effects that impact the decision making processes of customers in relation to donations. This potential shortcoming could be investigated by comparing results from campaigns whose topic overlap with the products on offer in-store with results from campaigns that do not overlap. Furthermore, there is also a need to see if our results can be replicated in different fundraising contexts, such as email or social media fundraising campaigns, as well as in different countries.

Our analysis suggests that research should focus on real-world campaigns rather than hypothetical survey data. Past work has shown that basing research on hypothetical donations can result in inflated donation estimates [20], but our results suggest more fundamental problems with this approach. This is because respondents in surveys of hypothetical preferences focus clearly on the task of choosing between flagships [32, 33], whereas in the real world they are often more distracted and so may make decisions based on different factors. This is not to say that potential donors to more active campaigns, such as giving to people collecting for charity on the street, would show a similar lack of discrimination between flagships. However, it does echo calls from other disciplines that caution is needed when interpreting results from studies that do not match the conditions under which people make decisions in real life [60].

### Fundraising insights

Our results show that campaign duration did not influence total revenue, thus rejecting our hypothesis. This may have been because individual customers visited the shops infrequently and so donated to new campaigns every time they visited or due to a strong seasonality effect, which meant that other factors where driving the amounts fundraised. The fact that the mean number of customers in the month before Christmas was more than four times the mean number of customers in the other months suggests that the latter might be the case. In this case the AGS would be free to adopt a more flexible approach to the duration of each campaign by, for example, running multiple campaigns simultaneously or by setting fundraising targets and changing the topic when these target amounts are reached. By changing from the current fixed term scheduling system, the AGS could pursue a more strategic approach and fundraise more for causes with higher costs.

The increase in donations over time we detected, even after correcting for inflation, should be noted as a positive sign for Australian charities. Yet, our finding that this variable had more explanatory power than the characteristics of the campaign or the traits of the potential donor population show how broader socio-economic trends can be critical to dictate the outcomes of fundraising initiatives. In Australia it is likely that the country’s economic growth over the last decade, which led to an increase in real net national disposable income per capita [38], enabled Australians to be progressively more charitable [31].

We rejected our fourth and fifth hypotheses, because the only significant difference in donor profile was that shops in lower-income areas received higher revenue per customer (Table 2). This result contradicts previous research that found no association between donor socio-economic profile and probability of donating or amount donated [61], although it should be noted that the detected effect of income was small. Past research has also found that people with lower incomes are more likely to donate to charities with a human focus, instead of those for causes related to animals or the environmental [62]. We accept that our findings may have resulted from a limitation with our donor income metric, which was based on the characteristics of people living around the shops, rather than on the donors themselves. This might have been compounded by our inability to distinguish donors from non-donors, which reduced the resolution of the data and might have obscured some of the underlying drivers of donations. However, it is also possible that poorer people genuinely gave higher donations, and there are examples of similar patterns from studies on charitable giving in other sectors [27–29]. Regardless, our results add to the literature that suggests donor levels depend on a range of factors whose influence varies with the cultural context, campaign focus and donor profile. It is worth noting that while the AGS may gain strategically from focusing their fundraising efforts on their own customers, as they are more likely to donate, it is also true that this focus on a self-selected subject pool limits how widely our results can be extrapolated.

### Expanding research on conservation marketing

While some NGO staff know that research on past fundraising campaigns can be key for informing future marketing efforts [63], there is little published research on fundraising for biodiversity conservation to inform the broader community. This gap may be related to the public perception that marketing by NGOs is inappropriately “corporate” [64] but it may also be because NGOs do not encourage their staff to carry out such research by failing to provide the necessary time, training or incentives.

One way of fulfilling the need for more marketing research in the conservation NGO sector would be to promote collaborations between academics and NGOs [see 65]. This would give academics access to large volumes of real-world data and the ability to ensure their research remains applied, while NGOs would benefit from working with researchers with advanced analytical skills and an understanding of relevant theory [66]. However, NGOs will have to be comfortable sharing proprietary data that may reveal strategic insights about the impact of their marketing strategies and they may have to compromise around higher academic requirements for data generation (e.g. allow for fundraising campaign topics to be randomized across shops instead of implemented uniformly across all outlets). Whilst academics will have to work with NGOs to develop research questions that have real-world relevance, accept the NGO’s data sharing constraints, and recognise that the data collected may not meet the most sensitive theoretical assumptions. We argue that it is a worthwhile endeavour because such collaborations could play a decisive role in making fundraising efforts for conservation and environmental NGO’s more successful. The present research is an example of such collaboration, with the NGO gaining insights on how to improve their fundraising strategy, while the research contributes to what is known about the drivers of conservation donations.

## Supporting information

S1 Fig Gantt chart illustrating in black the timeline of the fundraising campaigns by the Australian Geographic Society included in this study. Red bars indicate shorter *ad hoc* emergency fundraisers, those that were organized in response to unforeseen natural disasters.
(DOCX)

S1 Table The appeal of species used by the Australian Geographic Society as flagships for their fundraising campaigns, measured as mean rank across respondents. Lowest rank indicates higher appeal. For the projects were more than one species or life stage was represented in the marketing materials, a mean of appeal mean rank for both relevant photos was used.
(DOCX)

S2 Table The familiarity of species used by the Australian Geographic Society as flagships for their fundraising campaigns, measured as percent of respondents who recognise a given species. For the projects were more than one species or life stage was represented in the marketing materials, a mean of the percentage of respondents familiar with both relevant photos was used.
(DOCX)

S3 Table Model selection to investigate the importance of flagship characteristics on individual donor behaviour at ΔAICc < 4. (DOCX)

S4 Table Model selection to investigate the socio-economic and demographic characteristics of customers in the area surrounding a Australian Geographic Society shop at ΔAICc < 4. (DOCX)
