# Supplementary material for: Why do people donate to conservation? Insights from a ‘real world’ campaign
Source: PLoS One. 2018 Jan 25;13(1):e0191888. doi: 10.1371/journal.pone.0191888 (PMC5785011; doi:10.1371/journal.pone.0191888)
Supplement: S1 Fig — Red bars indicate shorter ad hoc emergency fundraisers, those that were organized in response to unforeseen natural disasters. (DOCX) [file pone.0191888.s001.docx]

# “Why do people donate to conservation? Insights from a ‘real world’ campaign” by Veríssimo et al. (2016) – Supporting Information


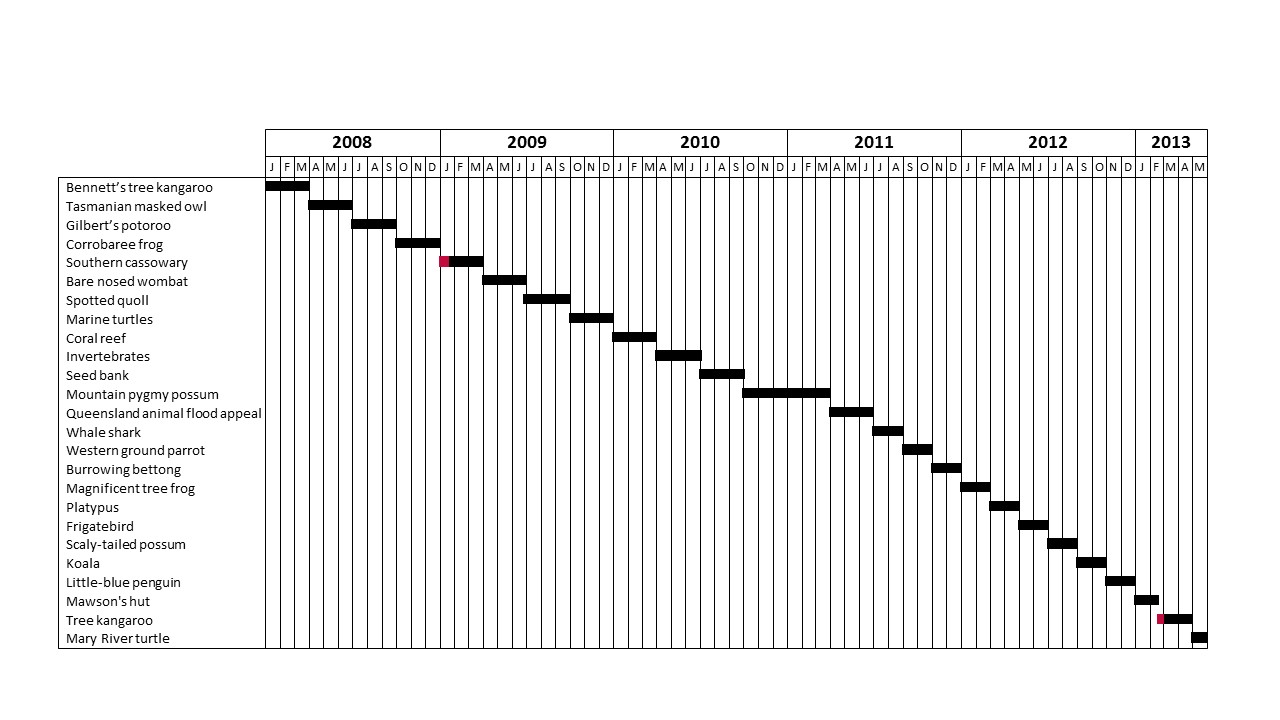


S1 Figure – Gantt chart illustrating in black the timeline of the fundraising campaigns by the Australian Geographic Society included in this study. Red bars indicate shorter *ad hoc* emergency fundraisers.
