# Supplementary material for: Why do people donate to conservation? Insights from a ‘real world’ campaign
Source: PLoS One. 2018 Jan 25;13(1):e0191888. doi: 10.1371/journal.pone.0191888 (PMC5785011; doi:10.1371/journal.pone.0191888)
Supplement: S1 Table — Lowest rank indicates higher appeal. For the projects were more than one species or life stage was represented in the marketing materials, a mean of appeal mean rank for both relevant photos was used. (DOCX) [file pone.0191888.s002.docx]

# “Why do people donate to conservation? Insights from a ‘real world’ campaign” by Veríssimo et al. (2016) – Supporting Information

S1 Table – The appeal of species used by the Australian Geographic Society as flagships for their fundraising campaigns, measured as mean rank across respondents. Lowest rank indicates higher appeal. For the projects were more than one species or life stage was represented in the marketing materials, a mean of appeal mean rank for both relevant photos was used.

| **Project focus** | **Species in marketing material** | **N** | **Appeal Sum Rank** | **Appeal Mean Rank** |
| --- | --- | --- | --- | --- |
| Platypus | Platypus | 52 | 160 | 3.1 |
| Common Wombat | Common Wombat | 43 | 161 | 3.7 |
| Sea Turtle | Sea Turtle hatchling | 51 | 194 | 3.8 |
| Spotted-tailed Quoll | Spotted-tailed Quoll | 49 | 201 | 4.1 |
| Sea Turtle | Sea Turtle Adult | 47 | 198 | 4.2 |
| Mountain Pigmy Possum | Mountain Pigmy Possum | 54 | 231 | 4.3 |
| Burrowing Bettong | Burrowing Bettong | 56 | 255 | 4.6 |
| Fairy penguin | Fairy penguin | 48 | 229 | 4.8 |
| Tree Kangaroos | Lumholtz's tree-kangaroo | 58 | 276 | 4.8 |
| Whale Shark | Whale Shark | 57 | 271 | 4.8 |
| Tasmanian Masked Owl | Tasmanian Masked Owl | 48 | 261 | 5.4 |
| Magnificent Tree Frog | Magnificent Tree Frog | 40 | 224 | 5.6 |
| Mary River Turtle | Mary River Turtle | 45 | 254 | 5.6 |
| Bennett’s Tree Kangaroo | Bennett’s Tree Kangaroo | 53 | 308 | 5.8 |
| Koala | Koala | 53 | 309 | 5.8 |
| Scaly-Tailed Possum | Scaly-Tailed Possum | 44 | 254 | 5.8 |
| Western Ground Parrot | Western Ground Parrot | 53 | 320 | 6.0 |
| Gilbert’s Potoroo | Gilbert’s Potoroo | 46 | 286 | 6.2 |
| Cassowary | Cassowary | 55 | 345 | 6.3 |
| Corroborree frog | Corroborree frog | 50 | 320 | 6.4 |
| Coral Reefs | Clown fish | 55 | 363 | 6.6 |
| Frigate bird | Frigate bird | 61 | 469 | 7.7 |
| Invertebrates | Katydid | 52 | 428 | 8.2 |
| Invertebrates | Emperor gum moth caterpillar | 40 | 338 | 8.5 |
