# Supplementary material for: Why do people donate to conservation? Insights from a ‘real world’ campaign
Source: PLoS One. 2018 Jan 25;13(1):e0191888. doi: 10.1371/journal.pone.0191888 (PMC5785011; doi:10.1371/journal.pone.0191888)
Supplement: S2 Table — For the projects were more than one species or life stage was represented in the marketing materials, a mean of the percentage of respondents familiar with both relevant photos was used. (DOCX) [file pone.0191888.s003.docx]

# “Why do people donate to conservation? Insights from a ‘real world’ campaign” by Veríssimo et al. (2016) – Supporting Information

S2 Table - The familiarity of species used by the Australian Geographic Society as flagships for their fundraising campaigns, measured as percent of respondents who recognise a given species. For the projects were more than one species or life stage was represented in the marketing materials, a mean of the percentage of respondents familiar with both relevant photos was used.

| **Project focus** | **Species in marketing material** | **N** | **Familiar** | **Familiarity %** |
| --- | --- | --- | --- | --- |
| Sea Turtle | Sea Turtle Adult | 47 | 47 | 100 |
| Platypus | Platypus | 52 | 52 | 100 |
| Fairy penguin | Fairy penguin | 48 | 48 | 100 |
| Koala | Koala | 53 | 53 | 100 |
| Common Wombat | Common Wombat | 43 | 42 | 98 |
| Coral Reefs | Clown fish | 55 | 54 | 98 |
| Cassowary | Cassowary | 55 | 52 | 95 |
| Sea Turtle | Sea Turtle hatchling | 51 | 48 | 94 |
| Magnificent Tree Frog | Magnificent Tree Frog | 40 | 35 | 88 |
| Whale Shark | Whale Shark | 57 | 49 | 86 |
| Spotted-tailed Quoll | Spotted-tailed Quoll | 49 | 41 | 84 |
| Corroborree frog | Corroborree frog | 50 | 41 | 82 |
| Tasmanian Masked Owl | Tasmanian Masked Owl | 48 | 39 | 81 |
| Mountain Pigmy Possum | Mountain Pigmy Possum | 54 | 42 | 78 |
| Mary River Turtle | Mary River Turtle | 45 | 33 | 73 |
| Western Ground Parrot | Western Ground Parrot | 53 | 35 | 66 |
| Gilbert’s Potoroo | Gilbert’s Potoroo | 46 | 28 | 61 |
| Scaly-Tailed Possum | Scaly-Tailed Possum | 44 | 27 | 61 |
| Bennett’s Tree Kangaroo | Bennett’s Tree Kangaroo | 53 | 32 | 60 |
| Frigate bird | Frigate bird | 61 | 36 | 59 |
| Tree Kangaroos | Lumholtz's tree-kangaroo | 58 | 30 | 52 |
| Burrowing Bettong | Burrowing Bettong | 56 | 28 | 50 |
| Invertebrates | Katydid | 52 | 20 | 38 |
| Invertebrates | Emperor gum moth caterpillar | 40 | 11 | 28 |
