# Supplementary material for: Why do people donate to conservation? Insights from a ‘real world’ campaign
Source: PLoS One. 2018 Jan 25;13(1):e0191888. doi: 10.1371/journal.pone.0191888 (PMC5785011; doi:10.1371/journal.pone.0191888)
Supplement: S3 Table — (DOCX) [file pone.0191888.s004.docx]

# Veríssimo et al. (2016) – Supporting Information

S3 Table – Model selection to investigate the importance of flagship characteristics on individual donor behaviour at ΔAICc < 4.

|  | Intercept | Appeal | Familiar | Location | Log weight | Time | df | logLik | AICc | delta | weight | R2 |
| --- | --- | --- | --- | --- | --- | --- | --- | --- | --- | --- | --- | --- |
| 17 | 0.29 |  |  |  |  | 0.11 | 5.00 | 513.57 | -1017.05 | 0.00 | 0.76 | 0.15 |
| 25 | 0.29 |  |  |  | 0.05 | 0.11 | 6.00 | 513.42 | -1014.71 | 2.34 | 0.24 | 0.19 |
